# Coded environments: data-driven indoor localisation with reconfigurable intelligent surfaces

**DOI:** 10.1038/s44172-024-00209-0

**Published:** 2024-05-16

**Authors:** Syed Tariq Shah, Mahmoud A. Shawky, Jalil ur Rehman Kazim, Ahmad Taha, Shuja Ansari, Syed Faraz Hasan, Muhammad Ali Imran, Qammer H. Abbasi

**Affiliations:** 1 University of Essex, School of Computer Science and Electronic Engineering, Colchester, CO4 3SQ UK; 2 University of Glasgow, James Watt School of Engineering, Glasgow, G12 8QQ UK; 3 Directorate of Research Services, University of New England, Armidale, NSW 2350 Australia

**Keywords:** Electrical and electronic engineering, Computer science

## Abstract

Reconfigurable Intelligent Surfaces have recently emerged as a revolutionary next-generation wireless networks paradigm that harnesses engineered electromagnetic environments to reshape radio wave propagation. Pioneering research presented in this article establishes the viability of Reconfigurable Intelligent Surfaces-enhanced indoor localisation and charts a roadmap for its integration into next-generation wireless network architectures. Here, we present a comprehensive experimental analysis of a Reconfigurable Intelligent Surfaces-enabled indoor localisation scheme that evaluates the localisation accuracy of different machine learning algorithms under varying Reconfigurable Intelligent Surfaces states, antenna types, and communication setups. The results indicate that incorporating Reconfigurable Intelligent Surfaces can significantly enhance indoor localisation accuracy, achieving an impressive 82.4% success rate. Moreover, this study delves into system performance across varied communication modes and subcarrier configurations. This research is poised to lay the groundwork for implementing Reconfigurable Intelligent Surfaces-empowered joint sensing and communications in future next-generation wireless networks.

## Abstract (web summary)

Syed Tariq Shah and colleagues use multi-antenna reconfigurable surfaces to maximise the accuracy of wireless indoor localisation. They study the achievable performance improvement using pre-trained machine learning techniques.

## Introduction

Next-generation wireless networks (NGWNs) operating on fifth-generation and sixth-generation technologies employ high-frequency wireless signals that are typically in millimetre-waves and sub-millimetre-waves ranges^1^. In addition to providing high data rate communication services, NGWNs are increasingly offering localisation services due to their ubiquitous nature^2^. From the context of NGWNs, localisation refers to determining a user device’s precise location within a pre-defined space. Being able to provide precise localisation service is crucial because it not only supports new applications like improved security provisioning but also enhances user experience and network efficiency^3–5^. Network efficiency can be improved by optimising the utilisation of available scarce radio and energy resources given users’ location^6^. A number of other state-of-the-art applications and services, such as asset tracking, space and people management, augmented/virtual reality programs, etc., also require precise location of users^7,8^.

Dedicated networks can be used solely to determine users’ location in global or local space. The Global Navigation Satellite systems (such as GPS, GLONASS, etc.) can localise an active receiver anywhere in the world. However, most existing systems require a direct line of sight between space-borne transmitters (satellites) and land-based user devices to achieve high localisation accuracy. The precision of existing localisation systems degrades severally in cases where user devices are located in an indoor environment (high-rise buildings, shopping centres, sporting venues, etc.). In addition to demonstrating poor precision in such environments, the Global Navigation Satellite system requires several satellite signals to perform localisation, which is often time-consuming and energy-inefficient^9^.

Stakeholders from academia and industry have conducted and reported on extensive research on localisation in wireless networks^10,11^. Various heuristic and AI-based approaches have been proposed for precise localisation in indoor environments. The domain of indoor localisation encompasses two primary methodologies: active and passive localisation. Active localisation, which is the focus of our considered system, involves scenarios where the target (a user or a device) is equipped with electronic means to participate actively in the localisation process^10^. This method typically offers higher accuracy and control, as the target actively transmits or responds to signals, facilitating more precise localisation. On the other hand, passive localisation, also known as device-free localisation, operates without necessitating the target to carry any electronic devices^11^. It exploits ambient signals and environmental interactions, such as signal reflections or distortions, to localise entities. While passive localisation offers the advantage of non-intrusiveness and broader applicability, it often contends with lower accuracy and greater susceptibility to environmental variables. Our approach to RIS-assisted indoor localisation specifically leverages the active approach, capitalising on the controlled signal interactions and the advanced capabilities of RIS technology. This choice aligns with our objective to develop a system capable of high accuracy and integrating seamlessly with the active components of NGWNs.

In the contemporary landscape of indoor localisation technologies, two predominant categories emerge, namely geometric distance-based algorithms and signal-strength correlation techniques. The geometric distance-based category encompasses methodologies such as time-of-arrival, time-difference-of-arrival, and angle-of-arrival^12,13^. Time-of-arrival and time-difference-of-arrival, in particular, are grounded in the principles of wireless signal transmission time, necessitating triangulation from at least three base stations to pinpoint a location accurately^14,15^. While these methods have shown reasonable performance, they require a much higher degree of time synchronisation, a feat not easily attained^16^. On the other hand, signal-strength correlation techniques are primarily grounded in the position fingerprinting approach. The position fingerprinting approach, which obviates the need for base station localisation and time-angle measurements, presents a more feasible alternative for indoor implementations. This method is gaining popularity as the preferred choice for indoor positioning due to its simplicity, ease of measurement and cost-effectiveness^17^. However, while the fingerprinting approach offers notable advantages such as simplicity and cost-effectiveness, it also presents various challenges, such as a time-intensive training phase and a susceptibility to localisation accuracy loss in the face of environmental changes. The fingerprinting approach can be classified into channel state information (CSI)-based fingerprinting and Received Signal Strength Indicator (RSSI)-based fingerprinting.

The CSI-based approach provides finer-grained multipath profiling by capturing amplitude and phase measurements across orthogonal frequency division multiplexing (OFDM) subcarriers. In the offline training phase, CSI fingerprints are constructed by sampling CSI parameters like delay and fading at specific locations. The online localisation phase then employs algorithms such as k-nearest neighbour (KNN), support vector machines (SVMs), and deep neural networks to estimate user locations by matching observed CSI fingerprints^18–20^. Recently, channel charting approaches have also gained attention. Such schemes learn to map CSI to channel charts in a self-supervised manner^21^. While promising, the effectiveness of CSI fingerprints is closely tied to the quality of channel impulse responses; therefore, CSI profiles can get distorted under poor channel conditions, leading to reduced localisation accuracy. However, similar to RSSI fingerprinting, where a radio map is constructed in an offline training phase by sampling RSSI from different access points at marked locations, CSI fingerprinting can also benefit from data aggregated from multiple access points. It is possible to create a more robust and comprehensive fingerprinting dataset by utilising CSI data from various sources. This approach can help mitigate the impact of poor channel conditions on CSI accuracy. After successful fingerprinting, an online localisation phase matches the observed RSSI/CSI to the fingerprints in the radio map using algorithms like kNN and neural networks to estimate the user location^22^. Similar to CSI-based approaches, RSSI-based approaches can suffer considerably from multipath fading and signal fluctuations, and their performance highly degrades when the channel quality is poor. In the presence of multipath fading, channel fluctuations and other similar parameters, it is not possible to yield accurate position estimates by employing legacy network approaches.

In addition to the challenges associated with different localisation techniques highlighted above, the use of high-frequency (millimetre-waves and sub-millimetre-waves) signals inherently results in poor propagation characteristics, thereby increasing the complexity of using them in localisation services^23^. Also, the typical NGWNs have a restricted communication range, with a largely unpredictable and often beyond-control radio propagation environment between user devices. To cope with these inherent shortcomings, Reconfigurable Intelligent Surfaces (RIS) have recently emerged as a possible method to control various aspects of radio channels, such as reflection, refraction, and scattering^24^. With the help of RIS, NGWN operators can enhance their network footprint by controlling and reshaping the radio environment’s EM response at quite a lower cost. This can be done by dynamically adjusting the transmitted signals’ amplitude, polarisation, and phase parameters^25^. Since RIS can shape radio wave propagation and boost received signal strength^26^, it can also potentially enhance localisation accuracy by overcoming undesirable signal characteristics. More specifically, as mentioned earlier, techniques such as CSI and RSSI fingerprinting used in traditional networks often face signal fluctuations and multipath fading issues, particularly in weak channel conditions, resulting in inaccurate localisation. However, with the assistance of RIS, received signals can be optimised by intelligently configuring the antenna elements to overcome undesirable propagation effects. This, in turn, greatly improves the accuracy of localisation. In our particular context, due to RIS, the improved RSSI fingerprints could enable more precise matching to radio maps and estimation of user locations. Furthermore, RISs can enable efficient localisation in contexts where traditional systems falter, reducing infrastructure costs and power consumption^25^. Therefore, RIS is expected to be an integral part of NGWNs not only to improve communications between user devices but also to localise them accurately^23^.

Despite the growing interest in RIS-enabled systems, limited works have delved into RIS for localisation, with the majority relying solely on numerical simulations. A multi-user 3D passive positioning scheme using user equipment equipped with RIS is proposed in^27^. Relying on time-of-arrival analyses, a low-complexity algorithm that employs orthogonal sequences in RIS phase profiles for accurate localisation is studied. This proposed method aims to reduce interference in passive localisation systems, particularly with RISs not integrated into the fixed infrastructure. With the help of simulation results, localisation error is evaluated. It is shown that it reaches the theoretical Cramér-Rao lower bounds (CRLB), and < 1 meter positioning error bound (PEB) can be achieved around the transmitter. Likewise, the authors in^28^ explore the use of RIS in NGWNs for improving user equipment localisation. In their proposed architecture, RIS assists in both position and orientation estimation at the gNB. The study advances beyond traditional methods by employing multiple antennas with 3D beamforming capabilities and a general model for both near- and far-field localisation in 3D space. It considers both synchronous and asynchronous signalling schemes, analysing their impact on localisation error. Drawing on the derived CRLB for assessing localisation and orientation, the simulation results reveal the notable performance (i.e., up to PEB of 8 × 10^−4^ meter at 28 GHz band) of the proposed method across various scenarios. Moreover, in asynchronous signalling contexts, the scheme demonstrates high efficiency, with the phase design closely approaching the optimal phase design that minimises the CRLB. Similar to the explorations in^28^, the study in^29^ delves into the impact of hardware impairments (HWIs) on RIS-aided localisation in NGWNs. It emphasises the use of RISs to mitigate HWI effects on localisation accuracy, offering a detailed analysis of the Fisher Information Matrix (FIM) and a robust Maximum Likelihood Estimator (MLE) for multi-RIS scenarios. The paper underscores the critical role of RIS configuration and the number of elements in enhancing localisation performance under HWI conditions. It is concluded that across varying inter-RIS spacings, the PEB decreased from approximately 0.3 meter to less than 0.15 meter as the hardware imperfection diminishes.

The study^30^ explores RIS-enabled near-field localisation in obstructed LOS environments. It introduces a theoretical framework to optimise the RIS phase matrix to enhance average localisation accuracy in a specified area. A solution for effective target localisation is presented using the discretisation method and iterative entropy regularisation algorithm. It is shown that the localisation accuracy of the proposed method enhances with the CRLB improving from around 1 meter at − 20 dB SNR to below 0.1 meter at 20 dB SNR. Unlike previous studies, which consider generic scattering environments, the authors in^31^ have established that RIS greatly enhance wireless communications in rich scattering environments. Through experimental case studies, they demonstrate RIS’s ability (in a rich scattering environment) to reshape channel impulse responses for improved communication rates and leverage wave fingerprints for precise non-LOS localisation of non-cooperative objects. Similarly, in contrast to most simulation-based studies where perfect hardware is assumed,^32^ proposes a technique for optimising RIS configurations, considering real hardware limitations. Utilising a pre-characterised lookup table of reflection coefficients, their method evaluates performance in terms of beam fidelity and localisation error across different RIS control strategies. Simulations reveal the effects of hardware constraints on beam power and the emergence of secondary lobes, impacting the localisation performance in non-LOS conditions. It is concluded that the PEB for the proposed scheme rises from below 10^−3^ meters to approximately 10^−1^ meters as the RIS-user equipment distance increases from 2 to 16 meters. In^33^, an innovative RIS system is presented, which simultaneously performs wireless communications and target tracking. Smart beam tracking and wireless communication are realised using a dual-polarized RIS and a pre-trained artificial neural network (ANN). Convolutional neural networks (CNN), along with computer vision, are used to detect the locations of moving targets automatically. By performing a series of experiments, the work in^33^ shows that this approach can foster intelligent wireless networks and adaptive systems, thereby paving the way for unified target identification and radio environment tracking solutions. It is also shown that while tracking the moving object, the proposed approach offers reliable network coverage and achieves a stable Bit Error Rate (BER) of 10^−5^.

The specific use of RIS in mmWave technology is also of great interest and presents critical and unique challenges. In ^34^, Jiguang et al. have studied RIS-aided mmWave multiple input multiple output systems for joint localisation and communication. An adaptive RIS phase shifter design is proposed based on hierarchical codebooks and limited feedback. During the phase shifter update process, the combining vector at the mobile station is sequentially refined. Simulations demonstrate that the proposed method reduces the Mean Squared Error for position error from above 10^2^ meters to approximately 10^−1^ meters as the SNR increases from −30 dB to 5 dB. Correspondingly, Moustafa et al. in^35^ conducted experimental validations of RIS-aided mmWave indoor positioning, utilising a dedicated frequency-domain mmWave indoor channel sounding campaign for high-resolution multipath analysis. The study focuses on the impact of RIS-reflected components like delay, angle-of-arrival, and AoD on localisation, benchmarking results across various user equipment and RIS locations, and identifying practical limitations such as grating lobes and distance constraints. It is concluded that the positioning errors for users in various scenarios range widely, with median errors as low as 0.07 meters and Root Mean Squared Error values reaching up to 3.22 meters.

Moreover, in^36^, an inverse semantic-aware wireless sensing framework is proposed, leveraging RIS technology for efficient data compression and encoding. The framework distinguishes itself by employing a semantic hash sampling method, which surpasses traditional uniform sampling in efficiency. Additionally, it introduces a self-supervised decoding method capable of recovering signal spectrums without the need for pre-training. The experimental findings showcase a 95% reduction in data volume and a 67% lower Mean Squared Error in sensing parameter recovery. From a telemedicine perspective,^37^ explores a method that combines RIS with frequency-modulated continuous wave radar for indoor people monitoring. By directing the radar beam through RISs from different angles, the method improves the accuracy of locating multiple subjects in a two-dimensional space and reduces radar ghosting. In the experiments, RIS functions were simulated using manually rotated flat metal plates. The results demonstrate effective localisation of multiple subjects, with positioning errors ranging between 10.05 cm and 14.21 cm. Furthermore, in the field of RIS-enhanced telemedicine systems, a study in^38^ delves into intelligent indoor robotics aimed at smart healthcare. This work introduces an intelligent cyber-physical robotic system, which is empowered by programmable RIS and augmented by AI tools. Central to this concept is the integration of a robotic brain that performs complex sensing tasks, including the localisation of mobile robotic limbs and human posture recognition.

While the above-mentioned studies on RIS-aided localisation provide valuable insights, they largely focus on simulations and operate under certain assumptions, such as ideal RIS technology, controlled signal propagation, RIS integration in user equipment, and precise synchronisation. It’s important to note, however, that these studies might not fully account for real-world complexities like environmental variability, hardware imperfections, and dynamic user behaviour.

In this article, we introduce a RIS-enabled localisation system for indoor scenarios, particularly focusing on environments where the direct received signal strength (RSS) from the transmitter is poor. This work represents an important step in the practical application of RIS technology for improving localisation in next-generation wireless networks. Our study successfully demonstrates the feasibility of using RIS for indoor localisation, achieving over 82% localisation accuracy. This suggests a promising direction for enhancing indoor positioning systems, highlighting the utility of RIS in such applications. We have developed a comprehensive experimental framework that includes various RIS states, antenna setups, and channel conditions, providing a thorough evaluation of the system’s performance under different scenarios. One of the key aspects of our research is the development and practical evaluation of a RIS localisation system. This effort contributes to bridging the gap between theoretical research and applicable solutions in the field. Alongside, we have rigorously evaluated the performance of different Machine Learning algorithms (such as Gradient Boosted Trees (GBT), Naïve Bayes, Random Forest, Support Vector Machine, Logistic Regression, Neural Networks,^18–20^ etc.), identifying effective approaches and hardware configurations for RIS-aided localisation.

**Fig. 1:**
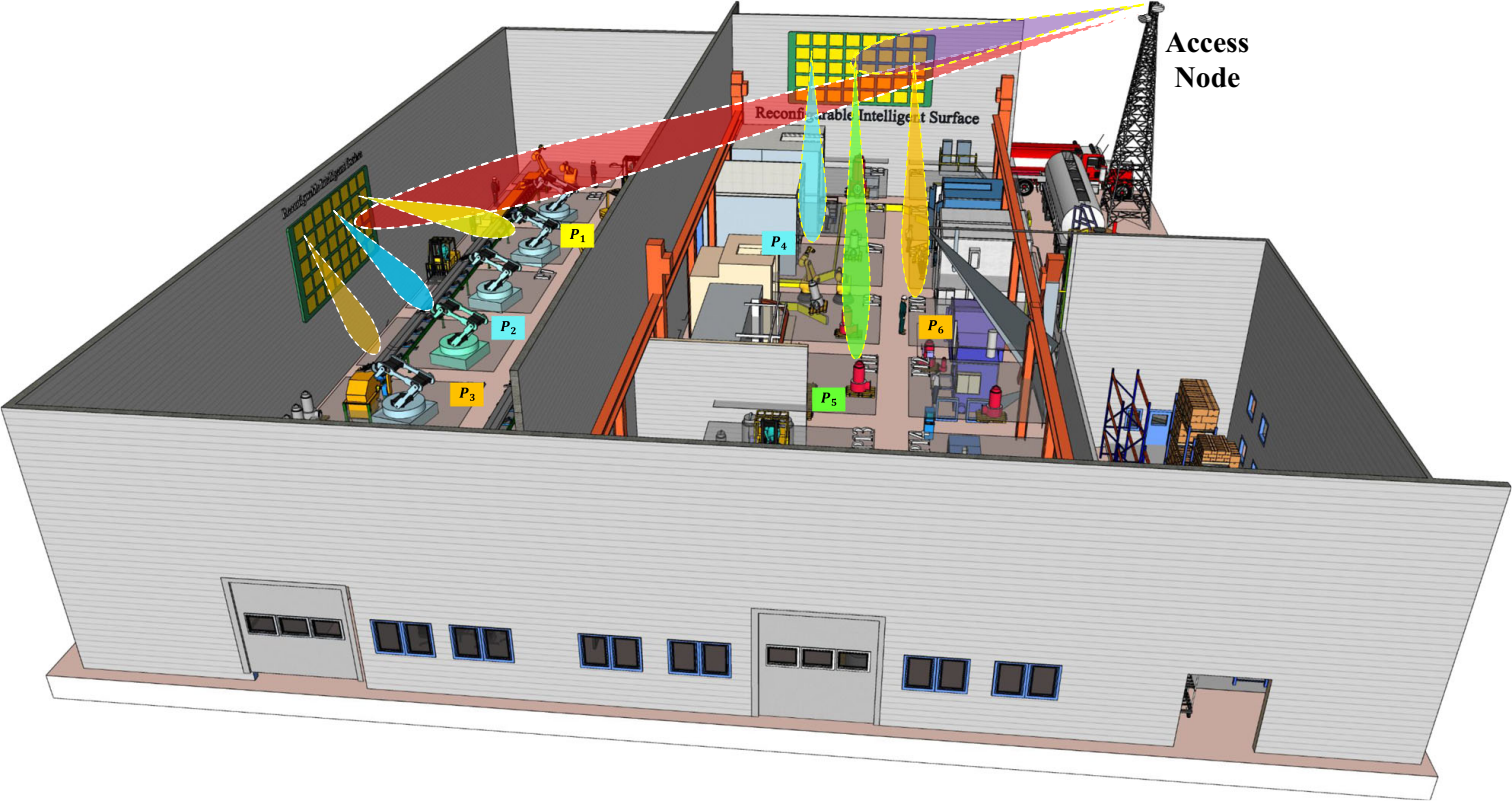
An example of RIS-enabled indoor localisation. A real-life visualisation of a reconfigurable intelligent surface (RIS)-enabled system efficiently localising the indoor target nodes.

Our detailed analysis provides insights into system performance across various communication modes, RIS states, and subcarrier setups. This helps in understanding the strengths and limitations of the system and highlights potential areas for improvement. Furthermore, our study examines the trade-offs in system robustness and performance, offering a realistic perspective on the challenges involved. By integrating RIS and localisation technologies, our work contributes to the evolving discourse on indoor positioning solutions. Figure 1 depicts a possible application scenario of our proposed indoor localisation mechanism in RIS-enabled NGWNs. It can be observed that the RIS can be exploited in an indoor environment not only to expand network footprint but also to improve localisation accuracy. Overall, this article contributes to the ongoing research in RIS for indoor localisation, offering insights and findings that can further enhance our understanding and application in this area.

## Methods

### Experimental setup and data collection

This section delves into the intricate details of the hardware-based experimental setup used to implement the proposed localisation scheme and collect the dataset mentioned above. The section also outlines the equipment, methodologies, and parameters employed, ensuring a comprehensive understanding of the process.

**Fig. 2:**
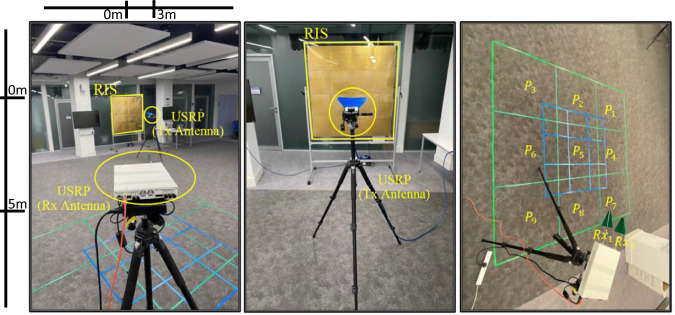
The designed experimental setup in operation. Operational view of the reconfigurable intelligent surface (RIS) based experimental setup, showing the access node as a universal software radio peripheral (USRP) transmitter (Tx) and the targeted node as a USRP receiver (Rx) in the localisation grid with different positions (P).

To prove the practicality of the proposed method, the experiment is conducted at the creativity lab at James Watt School of Engineering, University of Glasgow. As depicted in Fig. 2, the experimental setup consists of a 1-bit RIS with 64 × 64 elements and two universal serial radio peripherals version Ettus X300. The RIS is designed with 4096 elements, structured as 64 × 64 unit cells and segmented into 16 subarrays. Each subarray, measuring 33 × 33 *cm*^2^, comprises 256 p-i-n diodes and 16 × 16 unit cells, interconnected via 16-bit LED drivers in a serial daisy chain^26,39^. The overall RIS prototype spans 132 × 132 *cm*^2^ or 16.5*λ* × 16.5*λ* at 3.75 GHz. It’s mounted on a 142 × 142 *cm*^2^ polycarbonate sheet affixed to an aluminium frame. Each subarray has five interface lines for voltage, data, and connectivity, facilitating four subarrays per unit of daisy chain configurations. Data transfer to the RIS employs two SPI connections (SPI0 and SPI1) from a Raspberry PI 3B+ controller, clocked at 7.8 MHz. Over-the-air communication is achieved via a WiFi link between the MATLAB algorithm on a host PC and the Raspberry PI, which acts as a hotspot.

Moreover, the two universal serial radio peripherals act as a transmitter (*Tx*) and receiver (*Rx*). The transmitter is equipped with a single-directive antenna, featuring a 3 dB angular beamwidth of 80^∘^ in both the azimuth (E-plane) and elevation (H-plane). During the experiment, variations in the number of antennas on the receiver side ${N}_{R{x}_{ant}}=\{1,2\}$ are considered to present a single-input single-output (SISO) and single-input multiple-output (SIMO) communication system. Additionally, the experiment is conducted using directional UWB antennas at the receiver side to assess the impact of these parameters on the localisation accuracy. More specifically, the setup utilises log-periodic directional antennas as receiving antennas. These antennas have a frequency range spanning from 1.35 to 9.5 GHz, a standing wave ratio below 2.5, directional line polarization, and a gain ranging from 5 to 6 dB. Additionally, the experiment employs an OFDM communication system with varying numbers of subcarriers, *N* = {64, 128, 256}, and cyclic prefix lengths, *CP* = {16, 32, 64}. The carrier frequency is fixed at 3.75 GHz, with a sampling rate set to 200 KHz. On the receiver side, the experimental setup is divided into *T* = 9 positions, with two distinct inter-position spacings, *x* = {0.5, 1} m, as depicted in Fig. 2. Furthermore, the distance from the transmitter (*Tx*) to the midpoint of the RIS is 3 m, while the receiver (*Rx*) located at position 1 (*P*_1_) is positioned 5 m away. The transmitter’s antenna inclination is perpendicular to the RIS plane (*θ*_incident_ = 90^∘^), while the receiver’s antenna is angled at *θ*_reflection_ = 135^∘^. Both the transmitter’s and receiver’s antennas have a height of 126 centimetres, consistent with the midpoint of the RIS. The transmitter’s RF output power is 12.1 dBm. LabVIEW was employed to structure the OFDM symbols, with 256 subcarriers, of which 105 are allocated for zero-padding, 26 for channel probing and equalisation, 125 for data transmission, and 64 for the cyclic prefix. In this experiment, the data subcarriers are used for channel probing using reference symbols. The setup utilised the CBX-120 USRP daughterboard, offering a bandwidth of up to 120 MHz. The following summarises the steps involved in this experiment.

- *Step 1*: In the offline phase, the RIS is configured using Algorithm (1), generating nine optimum configurations ${H}_{j}^{{{{{{{{\rm{opt}}}}}}}}},\forall j=\{1,\cdots \,,9\}$, allocating the *Rx* at each *P*_*j*_.
- *Step 2*: The channel responses ($\hat{C{h}_{j}}=\hat{RS{S}_{j}},\hat{CP{R}_{j}}$) for each *P*_*j*_ have been estimated by transmitting a total of 1000 OFDM symbols from the transmitter (*Tx*) to the receiver (*Rx*). Each OFDM symbol comprises 256 subcarriers, out of which 125 subcarriers are dedicated to reference symbols utilised for estimating the received signal strength ($\hat{RS{S}_{j}}$) and the channel phase response ($\hat{CP{R}_{j}}$). This approach yielded 1000 received channel estimations, each containing 125 readings corresponding to the subcarriers in every OFDM symbol. Subsequently, the process was repeated with the RIS in both On and Off states, varying parameters such as ${N}_{R{x}_{ant}},x$, and *N* subcarriers. This rigorous procedure ensured a comprehensive dataset for training and classification purposes.
- *Step 3*: In this step, 80% of the channel estimates is allocated for training the ML algorithm, with the remaining 20% reserved for testing and evaluation.

For a comprehensive evaluation, we used different ML algorithms (see Table 1) to evaluate localisation accuracy at different parameter settings.

**Table 1:** Evaluation of classification accuracy across various applied machine learning algorithms

Experiment Setup	Method	Accuracy	Loss (cross-entropy)
Directive antennas, *x* = 1 meter distance, and reconfigurable intelligent surface (RIS) is activated	Gradient Boosted Trees	(82.4 ± 1.4)%	(0.547 ± 0.032)
	Naïve Bayes	(66.2 ± 0.5)%	(7.54 ± 0.21)
	Random Forest	(71.0 ± 0.5)%	(0.85 ± 0.34)
	Support Vector Machine	(80.4 ± 1.5)%	(0.586 ± 0.053)
	Logistic Regression	(78.5 ± 1.5)%	(0.655 ± 0.031)
	Neural Network	(26.9 ± 2.0)%	(13.3 ± 0.58)
	Decision Tree	(45.8 ± 1.5)%	(0.588 ± 0.039)
	Class Distributions	(64.2 ± 1.9)%	(1.87 ± 0.055)
	Nearest Neighbors	(61.2 ± 0.8)%	(1.12 ± 0.0092)

## Results

### Envisaged system architecture

**Fig. 3:**
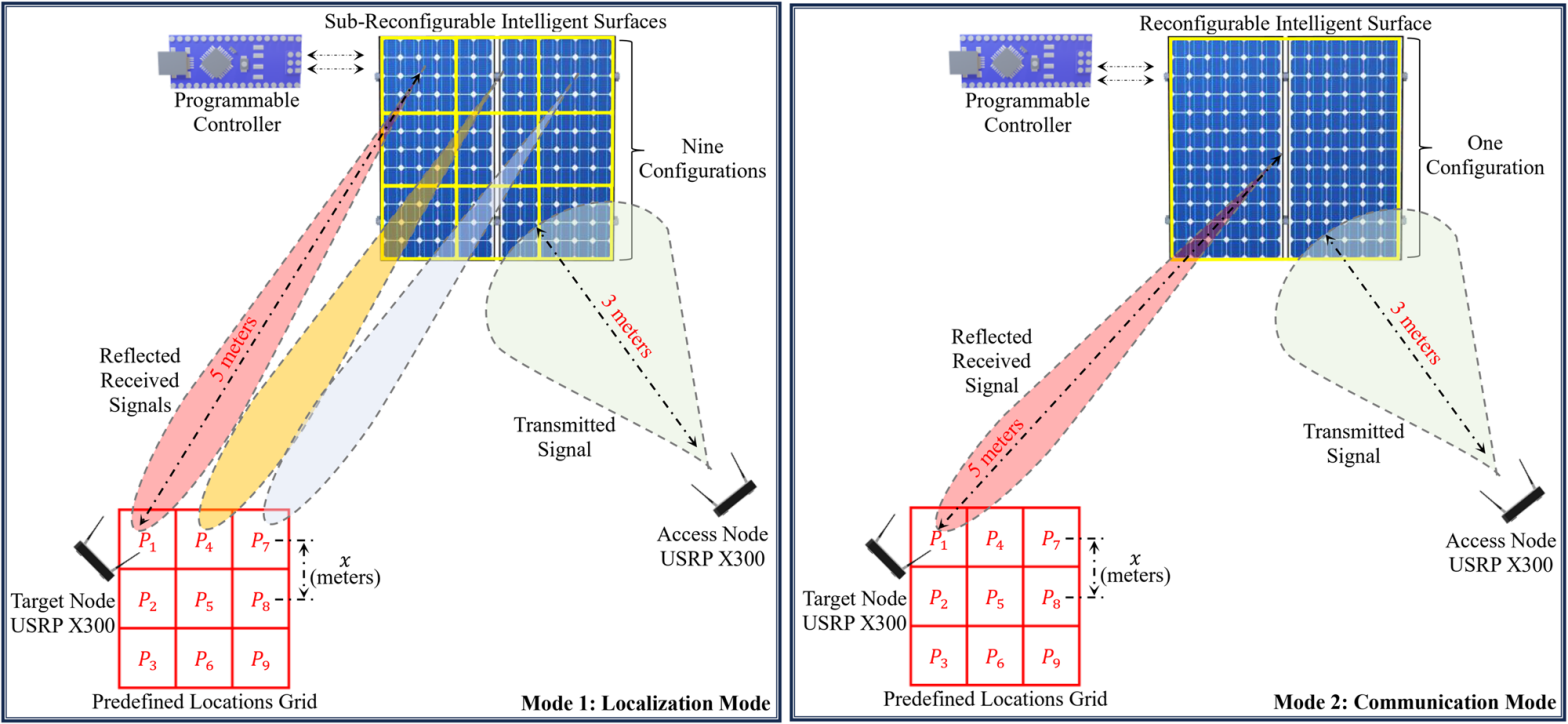
Illustration of the designed experimental setup. Depiction of the designed experimental setup, where both the localisation mode and the communication mode are shown. The terms USRP and P in the figure stand for universal software radio peripheral and different positions on the localisation grid, respectively.

The envisaged system architecture depicted in Fig. 3 comprises three major entities: the RIS, the target, and the access node. The functions of these entities are described in the following:

1. The RIS: The RIS in our envisaged system is a metasurface consisting of *N*_*x*_ × *N*_*y*_ number of reflective elements. It aims to enhance the wireless communication link between the target node (receiver) and the access point (transmitter). The RIS incorporates a programmable controller that allows it to intelligently manipulate its elements and amplify the signal strength at the desired receiving (target) node. In addition to improving and extending the communication range, the key objective of the RIS is to leverage its distinctive electromagnetic behaviour across different spatially (≥*λ*/2) separated target nodes to achieve efficient and accurate localisation.
2. The Access Point: The access point *A* acts as a central node, enabling communication between different network entities. It helps the network optimise RIS configuration by maintaining a reliable communication link with RIS’s programmable controller. Additionally, the access point is equipped with an offline-trained ML algorithm on the channel estimates obtained from different predefined and equally inter-spaced positions within the designated area of interest.
3. The Target: Targets refer to user devices intended for localisation by the network. A target node can be any device, such as a robot, IoT sensor, or power generator, actively communicating with the network and sharing information. Information signals received at these target nodes are essential in building an RF fingerprinting dataset, which is then utilised for target localisation with the help of ML. In this process, it’s crucial to consider various other noisy effects that can influence localisation accuracy, such as target mobility, interference, and multipath effects. Acknowledging and addressing these factors can further enhance the accuracy and robustness of the ML-based localisation method.

### RIS-Assisted Localisation

In this section, we introduce a robust RIS-assisted localisation scheme tailored for NGWNs. It is characterised by two modes of operation: target localisation mode and RIS-assisted communication mode. Subsequent subsections unfold a comprehensive technical discussion on each mode, offering an in-depth understanding of the intricacies involved in this innovative approach.

#### Target localisation mode

**Fig. 4:**
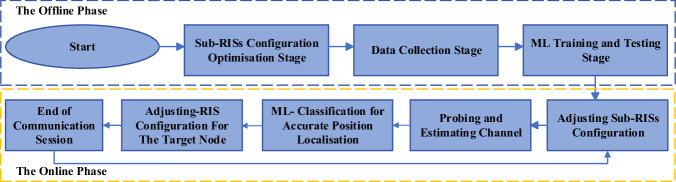
Flowchart of the proposed localisation approach. Schematic detailing the reconfigurable intelligent surface (RIS) configuration steps, from initial setup and data collection to real-time machine learning (ML)-based localisation and channel adjustment.

In this mode, the RIS, comprising a matrix of *N*_*x*_ × *N*_*y*_ reflective elements, is partitioned into a series of discrete sub-RISs *R*_*j*_, for *j* = {1, ⋯ *T*} with dimensions $\frac{{N}_{x}}{\sqrt{T}}\times \frac{{N}_{y}}{\sqrt{T}}$ elements, where the order of sub-RISs, denoted as ‘*T*,’ is contingent upon the prescribed level of localisation accuracy and the desired spatial resolution. Thus, a physical space spanning 3 × 3 *m*^2^ can be subdivided into 9 distinct positions denoted by *P*_*j*_, where *T* = 9, with an inter-position spacing, ‘*x*,’ of 1 *m*. Alternatively, for *T* = 18, the same area can be divided into 18 positions, with $x=\frac{1}{\sqrt{2}}m$. Selecting a smaller value of ‘*x*’ corresponds to enhanced localisation precision, whereas a larger value of ‘*x*’ results in coarser localisation resolution. Note that, in our experimental framework, each position *P*_*j*_ represents a true coordinate set, corresponding to the centre of the *j*^*th*^ subdivided area within 3 × 3 *m*^2^ physical space. Refer to Fig. 3 for an illustrative depiction of mode 1 within the RIS-enhanced localisation methodology. It can be observed that each sub-RIS *R*_*j*_ is uniquely associated with one of the *T* positions *P*_*j*_ within the grid, ensuring a direct one-to-one correspondence between the number of sub-RISs and the number of locations. Moreover, as depicted in the flowchart provided in Fig. 4, Mode 1 comprises two distinct phases: the offline phase and the online phase.

1. *The offline phase*: This phase serves as the initial configuration process for the localisation area of interest, considering the desired resolution ‘*x*’ and the acceptable localisation accuracy ‘*α*’. This phase encompasses the following key stages:
  *Sub-RISs configuration stage*: Given *x* and *α*, each sub-RIS *R*_*j*_ is configured in a way that maximises the signal-to-noise ratio (‘*SNR*_*j*_’) at position *P*_*j*_ from access point *A*. To facilitate this, we have devised a RIS configuration optimisation algorithm grounded in the principles of the Hadamard matrix codebook^40^. This innovative algorithm maximises the average *SNR*_*j*_ estimate derived from optimal configurations. The Hadamard matrix, which offers a range of orthogonal and binary phase shift values, can be applied to *R*_*j*_ elements, influencing the reflection patterns of incoming electromagnetic waves. We also define ${R}_{j}^{{{{{{{{\rm{opt}}}}}}}}}$ as the optimal RIS configuration for the (*j*^*th*^) sub-area that maximises the SNR.
  *Data collection stage*: In this stage, a number of *M* probing packets are employed by the access node to probe the channel between itself and the target node located at each position *P*_*j*_, ∀ *j* = {1, ⋯ , *T*}. This probing process yields estimates of the RSS and channel phase responses (CPR) estimates. This probing stage can be conducted for either a single input single output (SISO) system or a multiple input multiple output system for an OFDM communication system with *N* subcarriers.
  *Machine learning training and testing stage*: In this stage, 80% of the available *M* channel estimates are allocated to train the ML algorithm. This training process generates a set of distinct classes denoted as ‘*Cl*_*j*_’ for all positions within the set {1, ⋯  , *T*}. The remaining 20% of the *M* channel estimates are used to assess the proposed algorithm’s performance and generalisation for testing and validation purposes. It is during this crucial phase of training and validation that our system’s accuracy is established, which pertains to the success rate of correctly classifying a target’s location within predefined zones of the indoor environment. This classification accuracy, a key metric of our system’s effectiveness, measures how often the system accurately identifies the zone in which a target is located, relying on the RF fingerprinting data that is integral to our machine learning model.
2. *The online phase*: This phase is used for real-time localisation of a specified number of ‘*L*’ targeted nodes, denoted as ‘*n*_*l*_’, ∀ *l* = {1, ⋯ , *L*}, within the testing area. In this context, when ‘*A*’ intends to localise these target nodes, it broadcasts a localisation request. In response, each target node ‘*n*_*l*_’ replies with a probing packet, which is subsequently utilised for extracting the channel estimates. These extracted data are then employed in the ML classification algorithm to localise the target nodes accurately. Once the localisation process is successfully executed, *A* proceeds to select *n*_*l*_ with which it intends to establish communication. As explained below, communication between *A* and the intended device progressed in Mode 2.

#### RIS-assisted communication mode

The total RIS elements within this mode are systematically configured to optimise the RSS for the bidirectional communication link (*P*_*j*_ ↔ *RIS* ↔ *A*). The experimental setup designed for Mode 2 is depicted in Fig. 3. It is noteworthy that the optimisation process applied here follows the same procedure as outlined in Algorithm 1 for determining the optimal configuration of the RIS associated with each position ‘*P*_*j*_’, ∀ *j* = {1, ⋯ , *T*}.

##### Algorithm 1

Optimise Best Sub-RIS Configuration

**Require:** Sub-RIS (*R*_*j*_) with dimension $\frac{{N}_{x}}{\sqrt{T}}$ and $\frac{{N}_{y}}{\sqrt{T}},\forall j=\{1,\cdots \,,T\}$

**Ensure:** The best configuration for each Sub-RIS

1: Construct Hadamard codebook **HD**^1×*Q*^ as a collection of *Q* matrices:

2: 7D1**HD**^1×*Q*^ = {**H**_1_, **H**_2_, …, **H**_*Q*_}, where each ${{{{{{{{\bf{H}}}}}}}}}_{i}^{\frac{{N}_{x}}{\sqrt{T}}\times \frac{{N}_{y}}{\sqrt{T}}}$ is the *i*^*th*^ Hadamard matrix with dimensions $(\frac{{N}_{x}}{\sqrt{T}}\times \frac{{N}_{y}}{\sqrt{T}})$

3: Set $Q=(\frac{{N}_{x}}{\sqrt{T}}\times \frac{{N}_{y}}{\sqrt{T}})$ representing total reflecting units in *R*_*j*_

4: Initialise an empty matrix ${{{{{{{{\bf{SNR}}}}}}}}}_{{R}_{j}}=[]$ to store measured average SNRs

5: **for** each Sub-RIS index *j* from 1 to *T***do**

6: 7D1 Initialise ${{{{{{{{\bf{SNR}}}}}}}}}_{{R}_{j}}$ as an empty matrix

7: 7D1 **for** each matrix index *i* from 1 to *Q***do**

8: 7D17D1 Measure average SNR value $({\overline{{{{{{{{\rm{SNR}}}}}}}}}}_{i}^{{R}_{j}})$ for the *i*-th Hadamard matrix ${{{{{{{{\bf{H}}}}}}}}}_{i}^{\frac{{N}_{x}}{\sqrt{T}}\times \frac{{N}_{y}}{\sqrt{T}}}$

9: 7D17D1 Append $${\overline{{{{{{{{\rm{SNR}}}}}}}}}}_{i}^{{R}_{j}}$$ to $${{{{{{{{\bf{SNR}}}}}}}}}_{{R}_{j}}^{1\times (i-1)}$$

10: 7D1 **end for**

11: 7D1 Identify the best configuration ${{{{{{{{\bf{H}}}}}}}}}_{{R}_{j}^{{{{{{{{\rm{opt}}}}}}}}}}^{\frac{{N}_{x}}{\sqrt{T}}\times \frac{{N}_{y}}{\sqrt{T}}}$ that maximises the SNR value towards *P*_*j*_ and equals $Max\left({{{{{{{{\bf{SNR}}}}}}}}}_{{R}_{j}}^{1\times Q}\right)$

12: **end for**

**Return:** Best Hadamard matrix configuration ${{{{{{{{\bf{H}}}}}}}}}_{{R}_{j}^{{{{{{{{\rm{opt}}}}}}}}}}^{\frac{{N}_{x}}{\sqrt{T}}\times \frac{{N}_{y}}{\sqrt{T}}}$ for each Sub-RIS

## Discussion

This section analyses the plethora of data gathered through the meticulously conducted experiments outlined previously. The aim is to extract insights by dissecting the results and drawing comparisons to discern the most promising methods for localisation in NGWNs, particularly when RIS is incorporated.

### Classification accuracy across various machine learning algorithms

Central to our analysis is the assessment of the efficacy of various machine learning algorithms in the context of target node localisation. The primary metric of interest is classification accuracy, which provides an immediate sense of how well each algorithm can predict the correct location of a device or user based on the received signal characteristics. Another pivotal metric is the loss, which gives insight into the overall error associated with the predictions. A lower loss value indicates better model performance, whereas a higher accuracy percentage underscores the algorithm’s proficiency in correctly classifying the data points. Table 1 delineates the classification accuracy and loss for each machine learning algorithm employed under a specific experimental setup where directive antennas were used, the inter-position spacing was fixed at 1 meter, and the RIS was activated.

From Table 1, it is evident that the GBT algorithm emerges as the frontrunner in terms of accuracy, achieving an impressive 82.4 ± 1.4%. This high accuracy, coupled with a relatively low loss of 0.547 ± 0.032%, underscores the potency of GBT for localisation in RIS-assisted NGWNs. Despite its reputation for handling complex patterns, the Neural Network registers a meagre accuracy of 26.9 ± 2.0%, highlighting the challenges in deploying deep learning techniques without substantial data or in scenarios where simpler algorithms might suffice. Furthermore, given the experimental setup and the nature of the dataset, the GBT model’s ability to capture non-linear relationships, handle feature interactions, and its robustness to outliers have contributed to its superior performance compared to other machine learning models.

### In-depth evaluation of localisation accuracy based on antenna types, RIS activation and classification performance metrics

Antennas play a crucial role in any wireless communication setup, influencing the propagation characteristics of electromagnetic waves and, therefore, the received signal quality. In the realm of localisation, where the essence lies in deriving spatial information from these signals, the choice of the antenna becomes even more pivotal. In Table 2, we have conducted a comprehensive analysis to discern the impact of different antenna types on classification accuracy, using the GBT algorithm for localisation. Specifically, we have compared the results obtained using Directive and Monopole antennas, both in the presence of activated and deactivated RIS. Furthermore, we have considered two inter-position spacings: *x* = 1 meters and *x* = 0.5 meters. The results in Table 2 demonstrate clear benefits of RIS activation, consistently improving localisation accuracy across configurations. Specifically, directive antennas outperform monopole variants, as their focused propagation leverages RIS enhancements most effectively. However, reducing inter-position spacing degrades accuracy, given the more challenging granularity. Loss metrics align with accuracy trends, with lower losses in higher-accuracy scenarios - highlighting the consistency of RIS-enabled localisation improvements using directive antennas, albeit with reducing gains at finer granularities. More specifically, the analysis of Table 2 quantitatively highlights the trade-off between localisation accuracy and system resolution in RIS-activated environments. With an increase in resolution from 1 meter to 0.5 meters, the directive antenna exhibits a 10.1% decrease in localisation accuracy, decreasing from 82.4% to 72.3%. In contrast, the monopole antenna shows a slight reduction in accuracy of 1.0%. These results also demonstrate the directive antenna’s increased sensitivity to enhanced resolution and delineate the delicate equilibrium between achieving finer localisation granularity and sustaining high accuracy. This balance is crucial for optimising RIS-enhanced localisation systems where precision is paramount.

**Table 2:** Evaluation of classification accuracy across different antenna types (Horn - Monopole) using the Gradient Boosted Trees machine learning algorithm for x = 1 and x = 0.5 meters distance between target node locations

Method	Distance (*x*)	Antenna type	RIS status	Accuracy	Loss (cross-entropy)
Gradient Boosted Trees	1 meter	Directive	Deactivated	(63.3 ± 1.8)%	(1.06 ± 0.035)
			Activated	(82.4 ± 1.4)%	(0.547 ± 0.032)
		Monopole	Deactivated	(59.4 ± 1.8)	(1.2 ± 0.035)
			Activated	(69.8 ± 1.7)%	(0.953 ± 0.027)
	0.5 meter	Directive	Deactivated	(54.5 ± 1.9)	(1.28 ± 0.029)
			Activated	(72.3 ± 1.7)%	(0.943 ± 0.035)
		Monopole	Deactivated	(51.4 ± 1.9)	(1.4 ± 0.035)
			Activated	(68.8 ± 1.7)%	(0.896 ± 0.036)

As discussed earlier, localising the positions of nodes in a wireless network is a classification task where the primary goal is to predict the correct position of a node based on certain features or measurements. In such tasks, apart from accuracy, other metrics such as recall and F1Score provide a more comprehensive understanding of the model’s performance. Table 3 presents the recall and F1Score for different positions (from *P*_1_ to *P*_9_) using the Gradient Boosted Trees algorithm, with directive antennas and *x* = 1 meter distance between positions. From Table 3, several insightful observations can be made. There appears to be position-dependent variability in recall and F1Score, with some locations seemingly more challenging to localise accurately than others. For instance, *P*_2_ and *P*_9_ have high scores while *P*_5_ is lower. Additionally, positions with higher recall also tend to have higher F1Score, suggesting the predictions made for those positions are not just frequent but also precise. Finally, the generally high recall and F1Score values with RIS activation provide further evidence that activating the RIS improves overall localisation capability. In summary, the table highlights nuances in localisation performance across different positions and the benefits of RIS. Building upon this understanding, it is essential to mention that despite our system demonstrating significant accuracy in determining the general area of a target, the inherent quantisation error embedded in this approach must be acknowledged. More specifically, in the context of our approach, quantisation refers to the division of the indoor space into discrete zones, each uniquely represented in our fingerprint database. The granularity of these zones, defined by their size and separation, directly impacts the localisation accuracy. Opting for finer granularity enhances precision but, at the same time, increases the complexity and size of the fingerprint database, which could affect the system’s efficiency and scalability. On the other hand, coarser granularity simplifies the system but compromises the localisation precision. This trade-off in quantisation error is a fundamental aspect of our approach, balancing the granularity of the zones with practical considerations of system performance and scalability.

**Table 3:** For both reconfigurable intelligent surface (RIS) activated and RIS deactivated modes, the evaluation of the Recall and F1 score across different positions (P) using the Gradient Boosted Trees machine learning algorithm, directive antennas, and for *x* = 1 meter distance between positions is provided

Position	RIS is activated		RIS is deactivated	
	Recall	F1Score	Recall	F1Score
*P*_1_	0.79	0.772616	0.655	0.680519
*P*_2_	0.91	0.903226	0.41	0.404938
*P*_3_	0.86	0.841076	0.9	0.841121
*P*_4_	0.825	0.800971	0.58	0.552381
*P*_5_	0.615	0.664865	0.605	0.596059
*P*_6_	0.87	0.863524	0.52	0.551724
*P*_7_	0.885	0.885	0.845	0.830467
*P*_8_	0.7	0.717949	0.575	0.60686
*P*_9_	0.9	0.891089	0.95	0.966921

The confusion matrix in Table 4 provides granular insights into the classification performance for each position. The predominance of high diagonal values representing true positives underscores the model’s proficiency in distinguishing between positions, especially with RIS activation. For instance, *P*_1_ through *P*_9_ had correct predictions for 158, 182, 172, 165, 123, 174, 177, 140, and 180. However, certain positions exhibited more misclassifications, suggesting potential overlaps in signal patterns. More specifically, *P*_3_ was frequently confused with *P*_1_ and *P*_2_ (10 times each), *P*_8_ with *P*_1_ and *P*_5_ (14 and 19 times, respectively), and *P*_5_ with *P*_4_ (21 times) and *P*_8_ (16 times). Since *P*_5_ is in a central location and, due to its topological centrality, it is more susceptible to misclassification into adjacent zones. The unique challenge at *P*_5_ arises from its proximity to multiple neighbouring zones, which increases the likelihood of signal pattern overlaps. As a result, *P*_5_ shows a higher rate of being incorrectly classified as *P*_4_ or *P*_8_, as compared to other positions. In contrast, *P*_2_, *P*_6_, *P*_7_, and *P*_9_ demonstrated fewer misclassifications, indicating more distinct feature sets the model captures well. In summary, while the confusion matrix reveals nuances between positions, RIS’s overall accuracy remains high, reiterating its critical role in localisation and the potential for refinements to improve performance further.

**Table 4:**
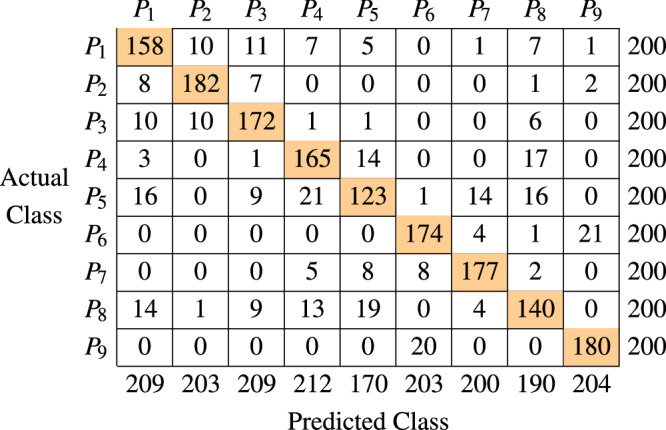
The confusion matrix of the proposed scheme using directive antennas at a distance of *x* = 1 meter between different positions (P), with the RIS in activated states

The highlighted cells in Table 4 represent the number of true positive predictions for each class.

**Fig. 5:**
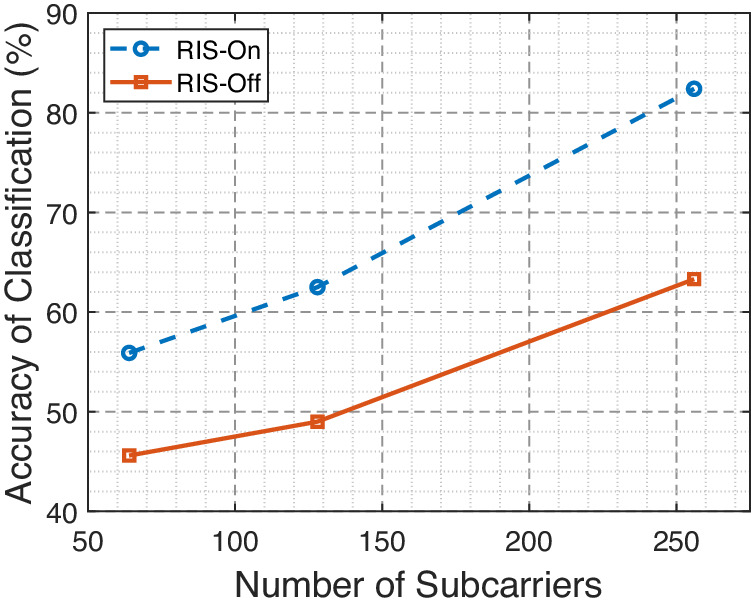
Impact of subcarrier numbers on the accuracy of classification. Classification accuracy across different subcarrier numbers (*N* = {64, 128, 256}) using directive antennas at a distance of *x* = 1 meter between different positions, with the reconfigurable intelligent surface (RIS) in both activated and deactivated states.

**Fig. 6:**
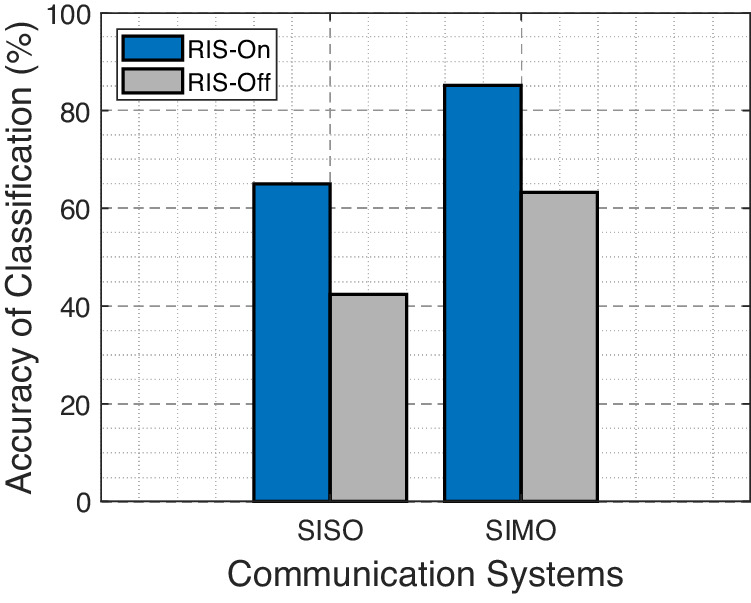
Impact of channel configurations on the accuracy of classification. Classification accuracy across different channel configurations, i.e., single input single output (SISO) and single input multiple output (SIMO), using directive antennas at a distance of *x* = 1 meter between different positions, with the reconfigurable intelligent surface (RIS) in activated and deactivated states.

Figure 5 depicts a performance comparison between the RIS-On and RIS-Off schemes in terms of classification accuracy across varying numbers of subcarriers. As evident, the RIS-On scheme consistently outperforms the RIS-Off scheme across all subcarrier counts. Notably, as the number of subcarriers increases, both schemes exhibit a trend of enhanced accuracy, with the RIS-On scheme maintaining a superior lead. Moreover, Figure 6 compares the RIS-On and RIS-Off schemes regarding classification accuracy for both SISO and SIMO communication modes. The RIS-On scheme consistently outperforms the RIS-Off scheme for both modes. Moreover, when transitioning from SISO to SIMO, both schemes show a notable increase in accuracy, highlighting the enhanced performance of the SIMO configuration.

## Conclusion

The study presents a pivotal exploration of RIS-enabled indoor localisation, demonstrating an integration of RIS technology with machine learning to enhance indoor localisation accuracy in next-generation wireless networks significantly. Through comprehensive experimental analysis, it is identified that integrating RIS with various machine learning algorithms, notably Gradient-Boosted Trees, can achieve up to 82.4% localisation accuracy. This study marks a significant advancement in the domain of indoor localisation, showcasing the innovative integration of RIS technology with machine learning to markedly improve indoor localisation accuracy within next-generation wireless networks. A thorough experimental analysis reveals that the fusion of RIS with various machine learning algorithms, especially GBT, facilitates achieving an impressive indoor localisation accuracy of up to 82.4 Further analysis shows the impact of different antenna types and communication setups on the localisation performance, providing insights into how these factors can be used to enhance accuracy further. Moreover, the research also shows an inherent trade-off between accuracy and granularity in localisation frameworks. The proposed approach, using classification for localisation, creates a discretised spatial model. Although effective, this approach has a limitation in achieving fine cm-level granularity compared to non-ML (geometric-based) localisation techniques.

## Data Availability

The authors declare that all relevant data are available in the paper or from the corresponding author on request.

## Code availability

The custom computer codes utilised during the current study are available from the corresponding authors on request.
